# Changes in Gallbladder Contractile Function and its Influencing Factors After Minimally Invasive Gallbladder-Preserving Surgery for Cholecystitis With Incarcerated Gallstones

**DOI:** 10.3389/fsurg.2022.926141

**Published:** 2022-07-14

**Authors:** Xiang-Nan Ai, Teng-Fei Wang, Qiang Zhang, Cai-Guo Ouyang, Zhen-Yu Wu

**Affiliations:** Department of Hepatobiliary Surgery, Aerospace Center Hospital, Beijing, China

**Keywords:** minimally invasive gallbladder-preserving surgery, cholecystitis with incarcerated gallstones, gallbladder contractile function, influence factor, gallbladder

## Abstract

**Background:**

Cholecystitis with incarcerated gallstones (CIG) is a type of acute abdomen in the field of hepatobiliary surgery. Whether gallbladder-preserving surgery (GPS) can be performed to treat it, however, depends on the improvement of gallbladder contractile function. The present study aimed to investigate the changes in gallbladder contractile function and its influencing factors after minimally invasive GPS for CIG.

**Methods:**

A total of 95 patients with CIG treated in the Aerospace Center Hospital between May 2017 and May 2019 were enrolled as the study subjects. All patients received minimally invasive GPS. The patients' operation-related conditions (including stone removal success rate, duration of surgery, intraoperative blood loss, etc.), changes in gallbladder contractile function, and influencing factors of GPS were analyzed.

**Results:**

Among the 95 patients included in the study, the success rate of stone removal was 100%, the duration of surgery was 76.0 ± 26.5 min, and the intraoperative blood loss was 10.17 ± 4.43 ml. The rate of good gallbladder contractile function at one and two years after surgery was significantly higher than before surgery (*P* < 0.05). Age, duration of surgery, stone recurrence, and diabetes were the independent risk factors for postoperative gallbladder contractile function (*P* < 0.05).

**Conclusion:**

Minimally invasive GPS for patients with CIG has a good curative effect. The changes in gallbladder contractile function after the surgery are influenced by many factors.

## Introduction

Cholecystitis with incarcerated gallstones (CIG), a type of acute abdomen in the field of hepatobiliary surgery, is a common form of cholecystitis. The main treatment for the disease is laparoscopic cholecystectomy. With the growth in understanding of the physiological function of the gallbladder and the development of choledochoscopy technology, the concept of gallbladder-preserving surgery (GPS) has been gradually accepted by physicians and patients, and the indications for GPS have slowly relaxed. However, the question of how to avoid recurrence has become the most important research direction for minimally invasive GPS. To date, a number of studies have confirmed that stone recurrence is related to dynamic gallbladder injury or gallbladder contractile dysfunction (1–3). As such, the present study enrolled 95 patients with CIG and treated them with minimally invasive GPS, after which any changes in gallbladder contractile function were observed and the factors affecting the recovery of gallbladder contractile function analyzed.

## Methods

### General Information

**Figure 1:**
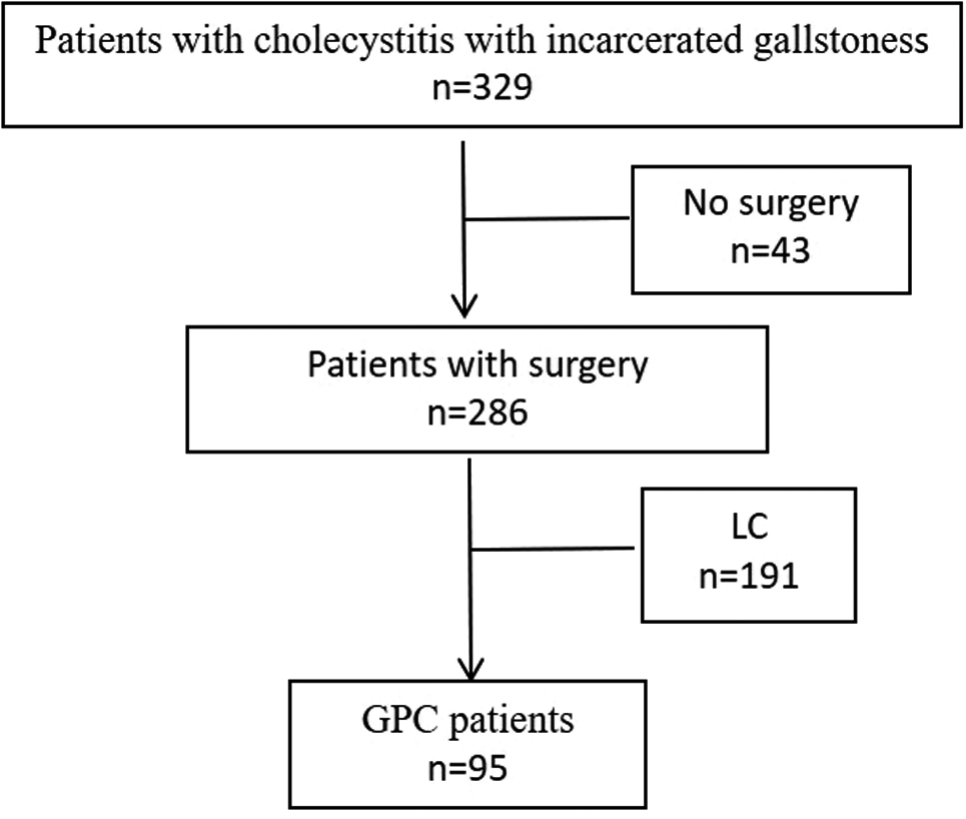
Flow chart of the exclusion of patients.

A total of 329 patients with CIG and a disease onset time of <48 h who were treated at the Aerospace Center Hospital from May 2017 to May 2019 were selected, 43 patients who did not undergo surgical treatment and 191 patients who underwent laparoscopic cholecystectomy for laparoscopic cholecystectomy were excluded. The remaining 95 CIG patients were included, including 36 males and 59 females, aged 25–74 years (Figure 1). All patients were definitively diagnosed with stone incarceration of the gallbladder neck or duct by imaging examination (B-ultrasound, computed tomography, etc.). The patient has the willingness and needs of gallbladder preservation. After treatment, the symptoms were relieved and the body temperature was normal. The gallbladder function test was performed to evaluate the preoperative gallbladder contractile function, which showed that there was no obvious edema of the gallbladder wall, and the plasma cholecystokinin level of each patient was normal. Patients with liver and kidney dysfunction, a history of upper abdominal surgery, acute attack of non-gallbladder inflammation, mental disorders, blood system diseases, etc., were excluded. The study was conducted with the informed consent of all patients.

### Surgical Procedure

**Figure 2:**
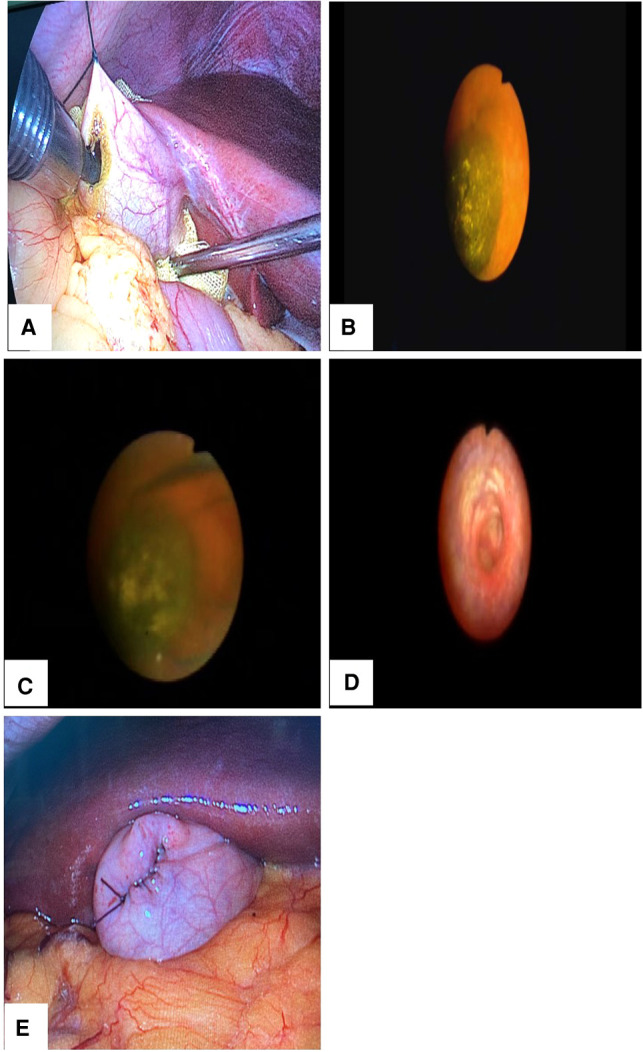
Intraoperative illustration. (**A**) The bottom of the gallbladder was suspended from the abdominal wall, and the choledochoscope was inserted into the gallbladder through a 12-mm disposable trocar. (**B**) Exploration found incarcerated gallbladder neck stones. (**C**) A stone extraction basket was used to remove the stone. (**D**) Exploration was undertaken to at least two spiral valves of the cystic duct after stone extraction to ensure that no stones remained and that the bile reflux was good. (**E**) The gallbladder incision was sutured with 4/0 absorbable sutures.

Minimally invasive GPS was conducted as follows: (1) The patient was placed in a supine position under general anesthesia, routine disinfection was performed, and surgical drapes were put in place. (2) An incision of 1.0 cm was made at the umbilicus, a 10-mm trocar was placed to establish pneumoperitoneum, and pressure was controlled within 10–12 mmHg. (3) A laparoscope was placed through the trocar, and the abdominal cavity was observed to determine the volume, location, appearance, and surrounding organs of the gallbladder. (4) A 5-mm trocar was placed under the xiphoid process, a 12-mm disposable trocar was placed under the costal margin of the right midclavicular line, and the bottom of the gallbladder was suspended on the abdominal wall. (5) The bottom of the gallbladder was incised with an electrocoagulation hook, a choledochoscope was placed *via* the 12-mm disposable trocar hole to explore the gallbladder and determine the location of the incarcerated stones in the gallbladder neck or duct, and corresponding methods were adopted to remove stones of different diameters (see Figures 2A–C). (6) The gallbladder exploration was continued, and 2–3 spiral valves in the gallbladder duct were observed to ensure that the stones were removed completely and that the bile reflux could be seen (see Figure 2D). (7) The gallbladder incision was sutured with 4/0 absorbable sutures, and the mucosal layer and seromuscular layer were sutured continuously (see Figure 2E), ensuring that there was no bile fistula or bleeding. (8) After surgery, the patients were treated with antibiotics, Danning tablets, ursodeoxycholic acid, and other drug therapies according to the doctor's advice. The patients were advised to take medication regularly for 6 months after surgery, and they were followed up regularly for two years.

### Observation Indexes

The patients' operation-related conditions, including stone removal success rate, duration of surgery, intraoperative blood loss, etc., were analyzed, and the gallbladder contractile function before and after surgery were compared. On this basis, the influencing factors for gallbladder contractile function were analyzed. To calculate maximum contractile rate of the gallbladder before and after surgery (i.e., the proportion of the difference between the fasting gallbladder volume and the minimum residual volume of the gallbladder to the fasting gallbladder volume). Three-dimensional ultrasound was used to measure the size of the gallbladder: after 6 h of fasting, the long, transverse and anterior-posterior diameters of the gallbladder were measured. After eating a standard fat meal (4), the measurement was repeated. The ellipsoid method was used, that is, gallbladder volume = 0.52 × long diameter * front and rear diameter * transverse diameter (5). Using this method, the recovery of gallbladder function was determined, with good function being indicated by a maximum gallbladder contractile rate of ≥50% and poor function being indicated by a maximum gallbladder contractile rate of <50%.

### Statistical Analysis

The SPSS 24.0 software was used for statistical analysis. Measurement data were expressed as mean ± standard deviation $(\bar{x}\pm \mathrm{SD})$ and compared using a *t*-test. Count data were expressed as a percentage (*n*[%]) and compared using a *χ*^2^ test. Univariate and multivariate logistic regression analyses were also carried out, with good gallbladder contractile function as the dependent variable and gender, age, and other factors as the independent variables. *P* < 0.05 was considered statistically significant.

## Results

### Analysis of Operation-Related Conditions

Among the 95 patients, there were 39 with more than five gallstones and 56 with five or fewer gallstones. The average gallstone size was 1.6 cm, with a range of 0.7–2.0 cm. There were 10 patients with diabetes (see Table 1). The success rate of stone removal was 100% (95/95), and none of the patients required conversion to laparotomy. The duration of surgery was 76.0 ± 26.5 min, and the intraoperative blood loss was 10.17 ± 4.43 ml. None of the patients experienced special discomfort after surgery. All patients undertook gentle exercise and ate liquid food on the first day after surgery, and they were hospitalized for two to four days, with an average of three days.

**Table 1:** Statistical analysis of general information of patients.

Variables	Points
Patients, *n*	95
Mean age, y (range)	38 (25–74)
Gender, *n*	
Female	59
Male	36
Gallstone number, *n*	
>5	39
≤5	56
Mean gallstone size, cm (range)	1.6 (0.7–2.0)
disease onset time, h	
≤24 h	62
24–48 h	33
History of cholelithiasis, y	1.5–10
With Diabetes	10

### Comparison of Good Gallbladder Contractile Function Before Surgery and One and Two Years After Surgery

The rate of good gallbladder contractile function was higher at one and two years after surgery than before surgery (*P* < 0.05; see Table 2).

**Table 2:** Comparison of good rate of gallbladder contractile function before operation and 1 and 2 years after operation (*n* [%]).

Group	Good rate of gallbladder contractile function (*n* = number of cases(%))
Before operation	15 (15.79)
One year after operation	48 (50.53)*
Two years after operation	78 (82.11)*

Notes: Chi-square test was used. Compared with before operation, *P < 0.05.

### Analysis of Factors Affecting Changes in Gallbladder Contractile Function

In the univariate analysis, duration of surgery, age, and diabetes mellitus were associated with postoperative gallbladder contractile function (see Table 3), and the differences were statistically significant (*P* < 0.05). In the logistic multivariate analysis, stone recurrence, age, diabetes mellitus, and duration of surgery were independent risk factors for the postoperative recovery of gallbladder contractile function (*P* < 0.05; see Table 4).

**Table 3:** Factors affecting the changes of gallbladder contractile function.

Group (*n* = number of cases)	Good gallbladder contractile function (*n *= 78)	Poor gallbladder contractile function (*n *= 17)	*χ* ^2^	*P*
Age				
>60 years old	4 (5.13)	7 (41.18)	17.716	0.000
≤60	74 (94.87)	10 (58.82)		
Gender				
Female	48 (61.54)	11 (64.71)	0.060	0.807
Male	30 (38.46)	6 (35.29)		
Number of stones				
>5	31 (39.74)	8 (47.06)	0.309	0.579
≤5	47 (60.26)	9 (52.94)		
Operation time				
>1.5 h	15 (19.23)	11 (64.71)	14.521	0.000
≤1.5 h	63 (80.77)	6 (35.29)		
Diabetes mellitus				
Yes	4 (5.13)	6 (35.29)	13.486	0.000
No	74 (94.87)	11 (64.71)		
Stone recurrence				
Yes	14 (17.95)	11 (64.71)	15.737	0.000
No	64 (82.05)	6 (35.29)		

*Notes: Univariate analysis was used (Chi-square test)*.

**Table 4:** Factors affecting the changes of gallbladder contractile function.

Factor	*β*	SE	WaLd	*P*	OR (95% CI)
Operation time	1.903	0.885	4.745	0.027	6.727 (1.309–26.451)
Diabetes mellitus	2.517	1.243	4.181	0.041	12.150 (1.070–39.017)
Stone recurrence	2.681	1.277	3.991	0.043	13.130 (1.157–33.511)
Age	2.430	0.471	26.065	0.001	11.260 (1.013–33.591)

*Notes: Logistic multivariate analysis was used.*

## Discussion

Gallstones can cause abdominal pain, fever, nausea, vomiting, and other symptoms; stones can stimulate the gallbladder wall for a long time and even carry the risk of canceration (6).

In the past, laparoscopic cholecystectomy was the gold standard for the treatment of gallstones. With the improvement of minimally invasive technology, however, choledochoscopy combined with laparoscopic minimally invasive GPS was proposed and widely applied. Furthermore, a study reported its advantages from multiple angles (7). Despite these advantages, the recurrence of gallstones is a significant problem related to minimally invasive GPS, causing patients a lot of worry. To solve this problem, many researchers have sought preventive measures for stone recurrence (8).

To date, several studies have reported a low recurrence rate of gallstones during long-term follow-up after minimally invasive GPS: Gao et al. conducted a long-term follow-up of 508 patients who underwent minimally invasive GPS, finding a gallstone recurrence rate of only 1.2% (9); Zha et al. reported 12-month, 36-month, and 60-month follow-up gallstone recurrence rates of 0%, 3.32%, and 5.64%, respectively, in 316 cases (10); and the Qu et al. found that most instances of recurrence occurred within two years of gallbladder-preserving cholecystolithotomy, with recurrence rates of 2.3%, 3.7%, and 7.6% at 6, 12, and 24 months, respectively(11). Regarding longer-term follow-up, the Liu et al. team reported that the five-year recurrence rate of minimally invasive GPS for stones in the neck of gallbladder was only 2.3% (12).

In the past, the gallbladder was not preserved during the removal of stones in the treatment of incarcerated stones in the neck or duct of the gallbladder; however, the incarceration of stones affects gallbladder contractile function, making it impossible to correctly evaluate the function of the gallbladder. This means that blind cholecystectomy requires the removal of the gallbladder in some patients with normal gallbladder function.

Patients whose stone incarceration time was <48 h and whose gallbladder wall had no obvious congestion or edema were enrolled in the present study, and minimally invasive choledochoscopic GPS was adopted to improve the stone clearance rate, alleviate symptoms, preserve gallbladder function, and improve quality of life. The results revealed that the success rate of stone removal in all patients was 100%, and the rate of good gallbladder contractile function was higher one and two years after surgery than before surgery (*P* < 0.05). These results suggest that minimally invasive GPS has good short-term and long-term outcomes.

There are many factors leading to gallbladder dysfunction (13). Combined with an in-depth analysis of relevant research, the results of the present study revealed that the recovery degree of gallbladder contractile function after surgery differs to a certain extent in different patients with cholecystitis. The altered gallbladder contractility may not only result from the inflammatory response within the gallbladder secondary to the stones but may also contribute to stone formation and cholecystitis (14). Actively mastering the factors affecting the recovery of postoperative gallbladder contractile function is therefore of great significance to its prognosis.

The results of the present study revealed that duration of surgery, age, diabetes mellitus, and stone recurrence are associated with postoperative gallbladder contractile function. On this basis, logistic multivariate analysis was carried out, and the results revealed that these factors were independent risk factors affecting the recovery of gallbladder contractile function after surgery (*P* < 0.05). There are several reasons for each of these independent risk factors:

Duration of surgery: Surgery that continues for too long not only increases the degree of trauma but also induces aseptic inflammation and edema of the gallbladder wall, which affects the recovery of gallbladder contractile function.

Age: Organ function gradually declines with age, resulting in low immunity. This prolongs the healing time of the gallbladder wall and increases the risk of cholecystitis recurrence after surgery, directly affecting the normal recovery of gallbladder contractile function.

Diabetes mellitus: The existence of an abnormal lipid metabolism, metabolic disorders, and other problems easily induces hyperlipidemia, which is a risk factor affecting gallstone formation. Furthermore, autonomic nerve injury, weakened gallbladder emptying function, gallbladder artery vascular disease, and other problems affect gallbladder emptying function (15).

Stone recurrence: Postoperative stone recurrence directly delays the recovery of gallbladder contractile function.

These results indicate that actively mastering the indications for minimally invasive GPS is the key to ensuring a good surgical effect and maintaining normal gallbladder contractile function in patients with CIG.

## Conclusion

Minimally invasive gallbladder-preserving surgery was performed on patients with stone-incarcerated cholecystitis, and the curative effect was definite. The changes of gallbladder contractile function after operation were related to the patient's age, diabetes mellitus, operation time and postoperative stone recurrence. Close attention to the above factors can improve the level of patient prognosis and recovery.

## Data Availability

The original contributions presented in the study are included in the article/Suplementary Material, further inquiries can be directed to the corresponding author/s.
